# Known risk factors of the developmental dysplasia of the hip predicting more severe clinical presentation and failure of Pavlik harness treatment

**DOI:** 10.1186/s12887-023-03935-0

**Published:** 2023-03-31

**Authors:** Vilma Lankinen, Mika Helminen, Karim Bakti, Jarmo Välipakka, Hannele Laivuori, Anna Hyvärinen

**Affiliations:** 1 Department of Pediatric Surgery, Turku University Hospital, Turku, Finland; 2 Faculty of Medicine and Health Technology, Tampere University, Tampere, Finland; 3 Faculty of Social Sciences, Health Sciences, Tampere University, Tampere, Finland; 4 Tays Research Services, Tampere University Hospital, Tampere, Finland; 5 Pihlajalinna, Tampere, Finland; 6 Faculty of Medicine and Health Technology, Center for Child, Adolescent, and Maternal Health Research, Tampere University, Tampere, Finland; 7 Department of Obstetrics and Gynecology, Tampere University Hospital, Tampere, Finland; 8 Institute for Molecular Medicine Finland (FIMM), Helsinki Institute of Life Science, University of Helsinki, Helsinki, Finland; 9 Medical and Clinical Genetics, University of Helsinki and Helsinki University Hospital, Helsinki, Finland; 10 Department of Surgery, Mehiläinen Länsi-Pohja Oy, Kemi, Finland; 11 Department of Otorhinolaryngology and Head and Neck Surgery, Oulu University Hospital, Oulu, Finland; 12 Clinical Medicine Research Unit, Medical Research Center, University of Oulu, Oulu, Finland

**Keywords:** DDH, Developmental dysplasia of the hip, Risk factors, Pavlik harness, Ortolani positivity

## Abstract

**Purpose:**

Developmental dysplasia of the hip (DDH) varies from mild instability of the hip to subluxation or total dislocation of the joint. Well-known risk factors of DDH include pre-natal breech position, female sex, positive family history, hip side, primiparity and the mode of delivery. Aim of the present study was to further evaluate known risk-factors of DDH, find associations with more severe dysplasia (characterized with Ortolani positivity) and find risk factors of failure of the Pavlik harness treatment.

**Material and methods:**

All children with the diagnosis of DDH treated in Tampere University hospital in the years 1998–2018 were retrospectively identified for the study and the data was collected from the medical records. Teratological dislocations (*n* = 3) were excluded from the analysis. Total of 945 patients were included.

**Results:**

Breech presentation was strongly associated with Ortolani positivity (*p *< 0.001). Breech presentation was not associated with ending up for spica casting and/or operative treatment (*p* = 0.291) despite the association with Ortolani positivity. Ortolani positivity (*p* = 0.002), positive family history (*p* = 0.013) and girl sex (*p* = 0.029) were associated with ending up for spica casting and/or operative treatment.

**Conclusion:**

Breech presentation seems to increase the risk of Ortolani positive DDH. However, these infants are likely to recover with initially started Pavlik harness treatment, as it was not associated with elevated risk for undergoing more robust treatments. Positive family history and girl sex are associated with the most severe cases of developmental dysplasia of the hip, and it may predispose to the failure of the Pavlik harness treatment.

**Table:** 

**What is known**: Well-known risk factors of developmental dysplasia of the hip include hip side (left), girl sex, breech presentation and positive family history. Other associated risk factors are first born birth, mode of delivery, oligohydramnion and other foot deformities.
**What is new**: Association between well-known risk factors and more severe dysplasia (Ortolan positivity) and possible risk factors predisposing to failure of the Pavlik Harness treatment.

## Introduction

Developmental dysplasia of the hip (DDH) is a congenital abnormality of the hip joint. The condition varies from the mild instability of the hip, to subluxation or total dislocation of the joint. The most serious form of DDH is rigid dislocation of the femur head. The incidence of DDH has varied in the previous studies. Approximately 1 of 1000 child is born with a dislocated hip, but all the mild forms of DDH included, the incidence has reported to be 1–7% of the newborns. [1] Even though some mild forms of DDH can resolve spontaneously, early detection of DDH is important. If not treated correctly in infancy, DDH can lead to difficult rigid luxation of the femur head, leading to long spica casting treatments or surgical operations and further frequently leading to early total hip replacement [1, 2]. The diagnosis of DDH is made by clinical examination after birth. Ortolani and Barlow tests are widely used among clinicians to describe the stability of the hip [1]. Ultrasound screening routinely after birth is not recommended, as it leads to overtreatment of the condition and does not help to avoid late detected cases of DDH. [3, 4] Instead, selective secondary screening by six weeks of age is widely used in children with known risk factors for DDH [5, 6]. However, the evidence of selective ultrasound screening is also inadequate and therefore there is no universal recommendations of ultrasound screening for hip dysplasia [7]. Ultrasound helps clinician to ensure the diagnosis, estimate the severity of the condition and monitor the development and growth of the hip joint during the treatment [6–8]

There are several known idiopathic risk factors of DDH. These risk factors include female sex, pre-natal breech presentation, positive family history, hip-side (left), primiparity and mode of delivery. [4, 9] The most important of these risk factors in meta-analysis were female sex, pre-natal presentation, side of the hip and positive family history [4]. Associations with post-maturity and higher birth weight have been reported, whereas prematurity has been associated with a decreased risk of DDH [10]. The most widely used method for treatment of DDH is Pavlik harness treatment, which has shown over 95% success rates in previous studies [11, 12].

## Aims

The aim of this study was to further evaluate risk factors of Ortolani positive DDH and identify potential risk factors in children who underwent spica casting and/or operative treatment. There are few studies about common risk factors for DDH or risk factors of late detection (over 3 months) of DDH [3, 4, 10, 13]. We did not find any studies evaluating risk factors specially associated with milder (Ortolani negative) or more severe (Ortolani positive) cases of DDH. Evaluation of these risk factors would give clinicians information of those infants that need special concern regarding on monitoring DDH and choosing for treatment. Several studies have identified risk factors for failure of the harness treatment, and it has been concluded that the reason for failure seems to be multifactorial [11]. These factors include more severe findings in the clinical evaluation (dislocation and irreducibility) [14, 15], more severe findings in initial ultrasound [15–19] and late detection of DDH (over 3–4 months of age) and late onset of treatment [19]. Bilaterality has also been identified as a risk for failure in some studies. [14, 20].

## Material and methods

All children with the diagnosis of DDH according to World Health Organizations International Classification of Diseases and Health Related Problems 9^th^ and 10^th^ revisions (ICD-9 and ICD-10), codes 7543.0–7543.5 (ICD-9) and Q65.0-Q65.5 (ICD-10), treated in Tampere University hospital in the years 1998–2018 were retrospectively identified for the study and the data were collected from their medical records. Those who received operative treatment in Tampere University hospital but were initially and postoperatively treated in some other location, were excluded from the study. Total of 948 patients were found. Three teratological dislocations were excluded from the further analysis.

### Population and our method

During the study period (20 years), 105 331 children were born in the Pirkanmaa region (Tampere University hospital) [21]. All newborn babies have their hips assessed. Suspected hip instability leads to further clinical examination by a pediatric surgeon at the age of 1–7 days. If Ortolani positive sign is detected, the treatment is started by Pavlik harness. Before the year of 2000, Frejka pillow was mainly used for the abduction treatment. Mild instability in clinical examination with or without Barlow positive sign, can lead to abduction treatment or watchful waiting depending on a clinician and parents’ opinion. All the children with hip instability undergo clinical and ultrasound examination at the age of 3–6 weeks. If residual dysplasia is detected in the ultrasound, the abduction treatment is continued/started. Clinical and ultrasound examination are repeated every 4–6 weeks during abduction treatment and in every 1–3 months in watchful waiting until normal findings by the age of 6 to 10 months.

### Statistical analysis

Bivariate analysis of risk factors and Ortolani positivity was carried out in cross-tabulations and Chi-square test was used to analyze statistical significance. In the second step, bivariate analysis of risk factors and undergoing spica casting or operative treatment were evaluated in cross-tabulations. Analysis of associations between birthweight and Ortolani positivity and between birthweight and casting/ operative treatment were made using T-test for comparing means. The statistical significance for all tests was set at *p* < 0.05. All of the analyses were carried out using SPSS version 23.

## Results

Total of 105 331 children were born in Tampere University hospital in 1998–2018. Of these children, 948 were diagnosed with DDH, of which 28 were late detected (over 3 months of age). Incidence of early detected DDH according to our data was 8.7/1000 newborns. Incidence of late detected DDH was 0.27/1000 newborns.

Teratological dislocations (*n* = 3) were excluded from the analysis. A total of 945 children were included. Majority (72.5%) were girls. Positive family history, defined as one or more cases of DDH among the first-degree relatives (parents or siblings), was reported in 587 children. Only 322 children had the information available on birth order in their medical records.

Ortolani negative DDH was diagnosed in 58.7% of the children and 30.9% had Ortolani positivity unilaterally and 10.3% bilaterally. Of 488 Ortolani positive hips, 316 (64.8%) were left sided and 172 (35.2%) right sided. Girls had Ortolani positive DDH (43.0%) more often than boys (36.5%), but the difference did not reach statistical significance (*p* = 0.072). Breech presentation was associated with Ortolani positivity (*p* < 0.001). In further analysis, it seemed that breech presentation increased bilateral Ortolan positivity (15.9%) compared to cephalic born infants (7.2%), *p* < 0.001 (OR 1.69 CI95% 1.28–2.23). When we further investigated the mode of delivery of the breech born infants, we found that caesarean section was strongly associated with Ortolani positivity (*p* < 0.001), whereas vaginal delivery was not (*p* = 0.417).

Preterm delivery (< 37^+0^ weeks of gestation) was associated with Ortolani negativity (*p* = 0.043), but prevalence of Ortolani positivity did not increase from term to postterm infants. Ortolani positive DDH was diagnosed in 21.6% of preterm, 42.4% of term and 40.9% of postterm infants, respectively. The other potential risk factors evaluated (first born, birthweight, positive family history) were not associated with Ortolani positivity. (See all risk factors in Table 1.)

**Table 1:** Risk factor analysis for Ortolani positive DDH

Risk factor (n)		Ortolan +	*p* value	OR (CI (95%))
**Gender**	F (684)	294 (43%)	0.072	
	M (260)	95 (36,5%)		
**Breech presentation**	Yes (309)	154 (49,8%)	< 0.001	1.692 (1.284–2.229)
	No (627)	232 (37,0%)		
**Firstborn**	Yes (201)	96 (47,8%)	0.490	
	No (121)	53 (43,8%)		
**Positive family history**	Yes (138)	58 (42%)	0.755	
	No (449)	182 (40,5%)		
**Pregnancy duration**	Pre-term (37)	8 (21,6%)	0.012	0.376 (0.170–0.833)
	Full-term (889)	376 (42,3%)		
**Birth weight**			0.171	

Birthweight was evaluated with T-test for comparing means. Mean weight in Ortolani positive children was 3526 g and 3474 g in Ortolani negative children. All the other comparisons are made using cross-tabulations with chi-square statistics

Forty-five children (4.8%) were treated with casting or/and operative treatment. Twenty-two children (48.9%) had the treatment with Pavlik harness before undergoing casting/operative treatment, eleven had 1 month duration of treatment, seven had 2 months duration and three had 3 months duration. One of the children initially treated with abduction did not have duration data. In 14 cases of 45 (31.1%) children, DDH was diagnosed over 3 months of age (late-detection). Nine patients had severe findings in their first ultrasound and/or clinical examination (alpha angle under 40, very small safety zone or severe abduction restriction in clinical examination), and were straight planned for operation and/or casting for that reason (no initial abduction). See the demographics about the children who underwent casting /operation in Table 2. Detection age was strongly associated with operative/casting treatment (*p* < 0.001). Risk for undergoing these treatments seemed to be elevated after 6 weeks of age (18.5%) and even higher after 3 months of age (50%). Ortolani positivity was associated with casting/operation *p* = 0.002 (OR 2.63 CI95% 1.39–4.90). Of the children with bilateral Ortolani positive DDH 10.3% were treated by casting/operation compared with 6.2% and 2.9% of the children with unilateral Ortolani positive and Ortolani negative DDH, respectively. Girls (5.7%) were more likely to undergo casting/operative treatment than boys (2.3%), (*p* = 0.029) (OR 2.56 CI95% 1.07–6.11). Children with positive family history of DDH (7.2%) were more likely to undergo casting/operative treatment than those without family history (2.7%), *p* = 0.013 (OR 2.85 CI95% 1.20–6.74). Birthweight (*p* = 0.371), breech presentation (*p* = 0.291), first born birth (*p* = 0.067) or preterm pregnancy (*p* = 0.794) were not associated with greater risk of undergoing casting/operative treatment, see all the results in Table 3.

**Table 2:** Patient demographics of the operatively/cast treated children

	**Age of patients at operation/casting**				
Months	1–3	3–6	6–12	12–36	over 36
*N* = 48 (%)	26 (54.2%)	2 (4.1%)	11 (22.9%)	6(12.5%)	3 (6.3%)
	**Age of patients at diagnosis**				
Months	< 1	1–3	3–6	6–12	over 12
*N* = 48 (%)	28(58.3%)	6 (12.5%)	1 (2.1%)	7(14.6%)	6 (12.5%)
	**Abduction treatment duration before operation/casting**				
Months	1	2	3	information missing	
*N* = 22 (%)	11 (50%)	7 (31.8%)	3 (13.6%)	1 (4.5%)

**Table 3:** Risk factor analysis for undergoing spica casting or operative treatment

Risk factor (n)		casting/operation	*p* value	OR (CI (95%))
**Ortolan + **	Yes (389)	28 (7.2%)	0.002	2.613 (1.394–4.899)
	No (555)	16 (2.9%)		
**Detection age**	Under six weeks (863)	26 (2.9%)	< 0.001	
	Six weeks to three months (27)	5 (18.5%)		
	Over three months (28)	14 (50%)		
**Gender**	F (685)	39 (5.7%)	0.029	2.556 (1.069–6.111)
	M (260)	6 (2.3%)		
**Breech presentation**	Yes (309)	11 (3.6%)	0.291	
	No (628)	32 (5.1%)		
**Firstborn**	Yes (202)	9 (4.5%)	0.067	
	No (121)	1 (0.8%)		
**Positive family history**	Yes (138)	10 (7.2%)	0.013	2.845 (1.201–6.737)
	No (449)	12 (2.7%)		
**Pregnancy duration**	Pre-term (37)	2 (5.4%)	0.794	
	Full-term (890)	40 (4.5%)		
**Birth weight**			0.853	

Birth weight and casting/operation were evaluated using T-test for comparing means. Mean weight in children who underwent casting/operation was 3472 g and 3496 g in children who did not. All of the other comparisons are made using cross-tabulations with chi-square statistics

In the group of children who underwent abduction treatment before casting/operation (*n* = 22), positive family history (*p* = 0.009) and Ortolani positivity (*p* < 0.001) remained statistically significant risk factors. In this population girls were also more likely (2.9%) to need operation (*p* = 0.050) compared to boys (0.8%). Other risk factors (first born birth, preterm pregnancy, breech presentation and birthweight) were not associated with higher prevalence of casting/operation in this population.

## Discussion

Incidences of early detected DDH and late detected DDH in our data, were comparable to resent meta-analysis, regarding the incidence of DDH in population with selected ultrasound screening. [22]

We found that the breech presentation was strongly associated with Ortolani positive DDH, and the risk of bilateral DDH was especially increased in breech born infants. The caesarean breech delivery was associated with Ortolani positive DDH in contrast to vaginal breech delivery. We also found that Ortolani positivity, positive family history and girl sex were associated with undergoing spica casting and/or operative treatment. Despite breech presentation associated with Ortolani positivity, it was not associated with undergoing casting/operation.

Breech presentation is a well documented risk factor of DDH [4, 10, 23]. More limited space in maternal pelvis for the infant in the breech position could explain the increased risk for Ortolani positive DDH. Our data suggests that C-section was associated with Ortolani positive DDH. It should be noted that this association stems from the infants’ breech presentation and that the C-section procedure itself does not increase the risk for Ortolani positive DDH. We hypothesize that these women have a narrower pelvis, therefore the infants breech presentation during development increases the risk. Our data found no connection with vaginal delivered breech presentation infants and Ortolani positive DDH. Thus, those mothers with wider pelvis might more often have vaginal deliveries even with breech presentation. Wider pelvis also gives more room for breech born infants, which might explain why there was no association with vaginal breech deliveries and Ortolani positivity. According to these findings, vaginal delivery can therefore be recommended for those mothers with favorable pelvis dimensions. Regarding to our findings, it is important for clinicians to give special concern to breech born infants with DDH, and start the treatment with Pavlik harness if needed. In our further analysis of winding up for operative or casting treatment, we found that breech position was not associated with increased risk of undergoing these treatments. This finding indicates, that even though breech position increases the risk of bilateral Ortolani positivity, these infants are likely to heal well with initially started Pavlik harness treatment and do not have elevated risk of undergoing casting/operative treatment.

In the present study prematurity seemed to be a protective factor from Ortolani positive dislocation. Smaller infants have more space in the pelvis and thus Ortolani positivity is not common. Our result is in agreement with some recent previous studies [24, 25]. There are also controversial results. An earlier meta-analysis by M. de Hundt et al. did not found association between prematurity and DDH, they included four previous studies on the issue. [23] However, more recent results are similar to ours indicating, that prematurity might be a protective factor of DDH.

In our data girls tended to have more often a more severe form of DDH (Ortolani positivity) compared to boys, all thought the association was not statistically significant. However, girls were more likely to need operative treatment compared to boys (*p* = 0.029). This finding might indicate, that in addition to sex being independent risk factor for DDH [23], it also might be independent risk factor for its more severe forms. Our findings are in contrast to the findings of some previous studies, in which an association was found between boys and failure of the harness treatment [16], although several studies have not found any association between sex and failure of the harness treatment [15, 18, 19].

In this study Ortolani positivity is associated with increased need of casting/operation in the children diagnosed with DDH. The risk seemed to increase with bilaterality. These findings could also be seen with the group of children who failed with initial treatment of Pavlik harness (*n* = 22). Dislocation and irreducibility in the initial clinical evaluation, together with severe findings in early ultrasound, has been associated with early failure of Pavlik harness treatment. Our findings further support these earlier studies. [11, 15, 16, 18, 26] Bilaterality has been associated with failure of the Pavlik harness treatment in some studies [14, 20, 27], and others have not found this association [28, 29].

It has been well reported that late started Pavlik harness treatment increases the risk of its failure and late diagnosis of DDH increases the risk of more robust treatments [19, 30–32] This can clearly be seen in our findings, which underline the importance of early detection of DDH and initially started Pavlik harness treatment to avoid casting/operative treatment. According to our findings, even the detection over 6 weeks (under 3 months) seems to increase the risk of these treatments, as 18.5% of these children needed surgery /casting in our data.

Positive family history is a well-known risk factor of DDH [3, 10, 23]. We did not find association between positive family history and Ortolani positivity, but the association was found between positive family history and operatively/ cast treated children, which suggests that genetic factors, could predispose especially on the most severe cases of DDH. Association of positive family history was also found in the group of children initially treated with Pavlik harness. This finding is new, and somewhat controversial compared to the findings of the previous studies reporting failure of Pavlik harness treatment [15, 18]. Despite the relatively small number of cases, especially the patients who failed the Pavlik harness treatment (22 patients), the association we made was strong. We think, that the reason why previous studies have not found association between positive family history and failure of the treatment is due to much smaller number of patients in these previous studies around the subject. [15, 18, 28]

Although the information was scarce of the birth order of children who underwent casting/ operation, first born birth had the trend to predispose to winding up for these treatments (*p* = 0.067). We did not find any previous studies with similar findings considering birth order as a risk factor for more severe DDH or failing of the Pavlik harness treatment. Meta-analysis from 2012 found, that first born birth is independent risk factor of DDH. [4] Our findings suggest, that first born birth could also predispose in more severe forms of DDH, although since our data was incomplete in this part, more research is needed to conclude this finding.

The present study has some limitations. Although the sample size was large, only 45 patients (4.8%) underwent casting/operative treatment. This was due to high success rate of Pavlik harness treatment combined with spontaneous recovery rate of the mild DDH. Because of the small number of these children, we did not further classify them to the subgroups of casting and operation. However, those children who initially required more robust treatment than Pavlik harness, were considered to have more severe DDH in general, thus our findings gave more information about the risk factors of having more severe form of DDH [11, 15].

We did not have early ultrasound screening protocol for all the patients with risk factors of DDH, rather we based our monitoring of DDH to findings of instability in the first clinical examination. Despite of that, our success rates for abduction treatments were excellent and in line with the previous research [11, 12, 18, 33]. In Tampere University hospital, the abduction treatment is initiated by pediatric surgeons or pediatric surgery residents only and the harness is worn 24 h/day in 2-week periods. In our protocol it was ensured, that the hips were reduced and harness was correctly initiated. We believe, that our great success rates of abduction treatment were due to this factor. Only 28 of late-detected cases of DDH (approximately 1,4 children annually) was found in our study which covered over 20-year period. According to our findings, the incidence of late detected DDH was 0.27/1000 newborns in our data. This is in line with the recent report from a Norwegian hospital with a selective ultrasound screening program [5] and with a retrospective study from United States with selective ultrasound screening program [6], as well as with resent meta-analysis of screening of DDH [22]. This indicates that our screening program is excellent without universal ultrasound screening.

The data was collected retrospectively, thus data available especially on family history and birth order were insufficient. This might affect the lack of association between Ortolani positivity and these factors. However, regardless of the small sample size and limited availability of information on these factors, the strong association was made between the positive family history and the need for casting/ operative treatment. In this study we did not further evaluate cumulative risk with those children with multiple risk factors for DDH. Further research is needed to evaluate and understand the cumulative risk of DDH.

## Conclusion

Breech position increased the risk of bilateral Ortolani positive DDH, however these infants had great potential of healing with initially started Pavlik harness treatment. Vaginal delivery in breech born infants did not further increase the risk of Ortolani positive DDH. However, caesarean section breech deliveries were significantly associated with Ortolani positivity, suggesting the breech presentation combined with reduced pelvic dimensions of the mother predisposing to Ortolani positive DDH. Our results suggest that positive family history and girl sex could be risk factors for failure of the Pavlik harness treatment. Still, prospective studies are warranted to confirm these findings.

## Data Availability

The datasets generated and/or analyzed during the current study are not publicly available due to patient privacy and confidentiality but are available from the corresponding author on reasonable request.

## Abbreviations

DDH: Developmental dysplasia of the hip
